# Microbial Community Succession and Organic Pollutants Removal During Olive Mill Waste Sludge and Green Waste Co-composting

**DOI:** 10.3389/fmicb.2021.814553

**Published:** 2022-02-21

**Authors:** Youness Bouhia, Mohamed Hafidi, Yedir Ouhdouch, Mohammed El Mehdi El Boukhari, Loubna El Fels, Youssef Zeroual, Karim Lyamlouli

**Affiliations:** ^1^Laboratory of Microbial Biotechnology, Agrosciences and Environment, Labelled Research Unit-CNRST N°4, Faculty of Sciences Semlalia, Cadi Ayyad University, Marrakesh, Morocco; ^2^Biodiversity and Plant Sciences Program, AgroBioSciences Department, Mohammed VI Polytechnic University (UM6P), Benguerir, Morocco; ^3^Situation Innovation - OCP Group, Jorf Lasfar, Morocco

**Keywords:** composting, metagenomic, microbial communities, organic pollutants, olive mill wastewater sludge (OMWS)

## Abstract

Olive mill wastewater sludge (OMWS) is the main by-product of the olive industry. OMWS is usually dumped in landfills without prior treatment and may cause several eco-environmental hazards due to its high toxicity, which is mainly attributed to polyphenols and lipids. OMWS is rich in valuable biocompounds, which makes it highly desirable for valorization by composting. However, there is a need to understand how microbial communities evolve during OMWS composting with respect to physicochemical changes and the dynamics of pollutant degradation. In this study, we addressed the relationship between microbial community, physicochemical variations and pollutants degradation during the co-composting of OMWS and green wastes using metagenomic- and culture-dependent approaches. The results showed that in raw OMWS, *Pichia* was the most represented genus with almost 53% of the total identified fungal population. Moreover, the bacteria that dominated were *Zymobacter palmae* (20%) and *Pseudomonas* sp. (19%). The addition of green waste to OMWS improved the actinobacterial diversity of the mixture and enhanced the degradation of lipids (81.3%) and polyphenols (84.54%). Correlation analysis revealed that Actinobacteria and fungi (*Candida* sp., *Galactomyces* sp., and *Pichia manshurica)* were the microorganisms that had the greatest influence on the composting process. Overall, these findings provide for the first time some novel insights into the microbial dynamics during OMWS composting and may contribute to the development of tailored inoculum for process optimization.

## Introduction

The world production of olive oil is estimated at 3 million tons annually, 98% of which are produced in the Mediterranean area (Souilem et al., 2017). The cultivation of olive trees is largely dedicated to oil and table olive production and has significant socioeconomic impacts (Uylaşer and Yildiz, 2014). However, the olive transformation industry generates large quantities of olive mill wastewater (OMWW), which is considered a serious environmental concern in Mediterranean countries (Galliou et al., 2018). Due to the complexity of OMWW, valorization strategies are scarcely implemented, and most industries often resort to storing byproducts in dedicated evaporation pounds (Jarboui et al., 2010; Kavvadias et al., 2010; Rajhi et al., 2018). Such a strategy, although more convenient in economic terms, is flawed because it induces the formation of olive mill waste sludge (OMWS), which is more hazardous than OMWW due to the higher load of pollutants (Kavvadias et al., 2017). The number of evaporation ponds throughout the Mediterranean area is continuously increasing, and their implication on the contamination of the soil and groundwater is frequently reported (Barbera et al., 2013; Mekki et al., 2013).

OMWS is a blackish-red solid material with complex and variable physicochemical characteristics. OMWS could be either acidic or basic depending on the olive quality, ripeness degree and extraction process (Rigane et al., 2015; Bouhia et al., 2020). Moreover, the stability of the generated sludge fluctuates significantly, as several aspects may affect the evaporation process, namely, the wind speed, light intensity and humidity of the area where OMWS is often dried for several weeks or even months depending on the weather conditions (Montero et al., 2015). Limited research has focused on the valorization of OMWS in comparison with OMWW. Successful examples include the production of construction materials (Hytiris et al., 2004) and the production of alternative fuels *via* pyrolysis of OMWS and waste tires (Grioui et al., 2019). With respect to biological methods, composting stands as a promising technology that has attracted the interest of scientific and industrial communities for the valorization of OMWS. Composting is one of the best approaches employed for the management of solid wastes and the production of stable soil amendments, which is perfectly aligned with the circular economy concept, as it fully integrates both economic and environmental aspects (Pergola et al., 2018). Nevertheless, due to the peculiarities of OMWS, composting remains intricate, and the use of classical approaches seems to be ineffectiveness in removing a large part of the existing organic pollutants (Hachicha et al., 2012; Sáez et al., 2021). Consequently, process optimization is required, which could be plausibly achieved by controlling the composting environment and/or microbial dynamics.

During OMWS composting, the variation in microbial community succession is dependent on the composting phase. The duration of each phase is dependent on the initial composition of organic material, moisture content, quantity, and composition of the microbial community. During the mesophilic phase, the initial decomposition of organic matter is ensured by mesophilic populations such as *Lactobacillus, Acetobacter, Penicillium*, *Aspergillus*, and *Streptomyces* by using readily available carbon sources at the beginning of the process (Chandna et al., 2013; Hefnawy and Nagdi, 2014; Li et al., 2019; Palaniveloo et al., 2020). As a result of mesophilic microorganism metabolic activity, the resulting heat leads to the transition to the thermophilic phase, where a high rate of biodegradation is recorded. In this phase, the growth of thermophilic organisms such as *Aspergillus, Bacillus*, *Actinobacteria, Thermoactinomyces*, *Talaromyces*, and *Pseudomonas* is observed (Chandna et al., 2013; Hefnawy and Nagdi, 2014; Li et al., 2019; Palaniveloo et al., 2020). However, it has been found that the most efficient composting process is achieved by both bacterial and fungal communities. The cooling phase, in which mesophilic microorganisms proliferate again in the compost, degrades the remaining organic matter. Many bacterial communities are associated with this phase, such as *Anthrobacter*, *Bacillus*, *Enterobacter*, and *Micrococcus*, as well as fungal genera such as *Alternaria*, *Aspergillus*, *Cladosporium*, *Trichoderma*, *Verticillium*, and *Zygorhynchus* (Palaniveloo et al., 2020). However, until today, there is a big lack of literature regarding OMWS microbiological structural community that are likely to play a major role during composting hygienization process. The objective of this investigation was to study microbial dynamics during OMWS composting by combining metagenomic and culture-dependent methodologies to pinpoint key microbial species and classes that may be involved in pollutant degradation to optimize the process and improve the quality of the final product.

## Materials and Methods

### Composting Assay

The co-composting assay was conducted in a bioreactor under controlled conditions. The feedstock was prepared by mixing equivalent quantities (20 kg) of OMWS and green waste (GW) consisting of grass and follicular parts of gardening work (Table 1).

**TABLE 1:** Physicochemical characteristics of OMWS (olive mill waste sludge) and GW (green waste): values are means ± SD of 3 replicates.

Parameters	OMWS	Green waste
Moisture^a^ (%)	53.11 (4.8)	57.64 (0.7)
pH^b^	4.9 (0.01)	7.15 (0.08)
EC^b^ (ms.cm^–1^)	3.72	0.73 (0.08)
TOC^a^%	50.18 (0.14)	47.38 (0.04)
TKN%	0.22 (0.01)	2.15 (0.29)
C/N	228	22
Total phosphorus (%)	0,126 (0,001)	0.04 (0.02)
Exchangeable potassium K^a^%	1.36 (0.01)	0.22 (0.02)
Exchangeable calcium^a^%	0.58 (0.01)	0.34 (0.01)
Exchangeable Mg^a^%	0.16	0.08 (0.01)
Total Cu^a^ (ppm)	20.58 (0.52)	2.91 (0.62)
Total Mn^a^ (ppm)	35.76 (0.36)	38.49 (3.18)
Total Fe^a^ (ppm)	1563.25 (38.81)	800.42 (156.9)
Total Zn^a^ (ppm)	58.86 (3.37)	24.24 (0.8)

*^a^Results expressed per unit weight fresh matter.*

*^b^Results expressed per unit weight dry matter.*

The mixture was homogenized, its moisture level was adjusted between 50 and 60%, and then the mixture was introduced into a 100 L, cylindrical bioreactor in stainless steel, with a superficial layer of insulating polyurethane equipped with a brewing system and air input and output to ensure uniform aeration during the experiment. At each stage of the composting process, 1 kg of the mixture was sampled and stored at -20°C, notably at T0 (initial mixture), T4 (after 4 days of composting), T9 (after 9 days of composting), T21 (mixture recovering from the bioreactor, after 21 days), T52 (after 52 days of the composting process; maturation in perforated bags) and T120 (after 120 of the composting process; maturation in perforated bags). The resulting compost product was equal to 14 kg.

### Chemical and Physicochemical Analysis

The composting temperature evolution was measured 4 times per hour during the whole process using sensors with data memory (PH0700115 Model 1.20 Ector-traceability software, ECTOR, France). For each sample during the composting phases, moisture was estimated by drying a sample at 105°C and measuring the weight difference. The pH and electrical conductivity (EC) were measured in an aqueous extract at ambient temperature from a 1/10 mixture of compost and distilled water. The total organic carbon (%TOC) was dosed by organic matter oxidation using K_2_Cr2O_7_ according to Walkley and Black (1934). The ash content was calculated after calcination at 600°C for 6 h. Total nitrogen (TKN) was assayed with 0.5 g dried samples by using classical Kjeldahl distillation according to AFNOR methods.

Phenols were measured based on the water extracts, successively treated with petroleum ether and ethyl acetate in the presence of ammonium sulfate and phosphoric acid, and the polyphenols were recovered with pure methanol. Quantitative measurements were carried out through colorimetric methods according to Box (1993) using Folin and Ciocalteu’s reagent at 760 nm. Lipid content was directly extracted from mixture samples using a dichloromethane/methanol solution with a Soxhlet apparatus and then evaporated to dryness under partial vacuum (Filippi et al., 2013; Bouhia et al., 2021).

### Phytotoxicity Analysis

Phytotoxicity was evaluated according to Zucconi et al. (1981) and Paulino et al. (2006) with slight modifications. Four grams of dry material from each composting phase was moisturized to 60%. Then, a volume of 50 ml of distilled water was added after 1 h, and the mixture was shaken for 2 h. The supernatant was recovered and filtered through a 0.45 μm Whatman membrane. Twenty seeds of each *Lepidium sativum* L. (Cress) and *Brassica rapa* (Turnip) were placed in petri dishes prior to the application of the filtrate. Seed germination and root elongation were evaluated. Tree replicates per sample were incubated in darkness for 48 h at ambient temperature. The germination index (GI%) was calculated with following the equation GI = [(GSs% × LRs)/(GSw% × LDw)]. Here, LRs: length of roots (mm) in the presence of the sample; GSs: number of seeds in the presence of the sample that germinated; GSw: number of seeds treated with water (control); and LDw: length of roots (mm) in seeds treated with water (control).

### Microbial Analysis

#### Cultivable Microbiota

Microbiological analysis of the indigenous cultivated microbiota was performed at different composting stages (0, 4, 9, 21, 52, and 120 days). After mixing, 1 g of substrate was taken and suspended in 10 ml of sterilized physiological water (9 g NaCl/L distilled water). The suspension was homogenized by first shaking and then treatment for 10 to 15 min by sonication (El Fels et al., 2015; Bouhia et al., 2021). Afterward, the suspensions were serially diluted to 10^–9^. The pH of each co-composting sampled mixture was adjusted to be the same.

For Actinobacteria enumeration, actinomycete isolation agar (AIA) (Sigma) was supplemented with 50 μg/ml cycloheximide and 10 μg/ml nalidixic acid to inhibit fungi and gram-negative bacteria without affecting the growth of Actinobacteria. Bacterial community enumeration was performed using standard medium (nutrient agar) with the addition of cycloheximide (Sigma). For estimation of the fungal community, suspension calibration was performed in potato dextrose agar (PDA) (Panreac) by the addition of 5 μg/ml chloramphenicol. Colony forming units (CFU) were counted in triplicate, and the results are expressed as CFU g^–1^ dry weight. The pH of each medium was adjusted to 5.6; 5.7; 5.9; 6.5; 7.4; and 8, respectively, for 0, 4, 9, 21, 52, and 120 days to be similar to that of each composting phases. Samples were incubated at both mesophilic (35°C) and thermophilic (45°C) temperatures.

#### Metagenomic Approach: DNA Extraction, Gene Amplification, and Sequencing

To study the variation in the total microbiota (bacteria and fungi) during the co-composting phases (mesophilic, thermophilic and maturation phases), we performed comprehensive metagenomic analysis. DNA from the samples (≈ 1 g of compost from each phase) was extracted by using a DNeasy PowerSoil Pro Kit (Qiagen Inc.) following the manufacturer’s instructions. Then, the quantity and quality of the extracted DNA were evaluated spectrophotometrically using a NanoDrop 2000c before being processed for 16S and ITS amplicon sequencing. Sequence libraries were generated using the complete primers ITS1 (5′CTTGGTCATTTAGAGGAAGTAA 3′) and ITS2 (5′ GCTGCGTTCTTCATCGATGC 3′) for fungi. For bacteria, the 16S rRNA gene V4 variable region PCR primers 515/806 were used in a 30–35 PCR cycle using HotStarTaq Plus Master Mix Kit (Qiagen Inc.). PCR was performed under the following conditions: 95°C for 5 min, then 30–35 cycles at 95°C for 30 s, followed by 53°C for 40 s and 72°C for 1 min. Finally, a final elongation was performed at 72°C for 10 min. Afterward, samples were multiplexed using distinctive dual indices and equally pooled together based on their molecular weight and DNA concentration. Then, they were purified using calibrated Ampure XP beads and used to prepare an Illumina DNA library. Sequencing was performed with MR DNA (Shallowater, TX, United States)^1^ on a MiSeq following the manufacturer’s guidelines, and sequence data were processed using MR DNA analysis pipeline (MR DNA, Shallowater, TX, United States). Briefly, sequences were joined, and sequences < 150 bp or with ambiguous base calls were removed. Sequences were then quality filtered (error threshold of 1.0) and dereplicated. The dereplicated or unique sequences were denoised, and unique sequences identified with sequencing and/or PCR point errors were removed, followed by chimera removal. thereby providing a denoised sequence or zOTU. Final zOTUs were taxonomically classified using BLASTn against a curated database from the NCBI.^2^

#### Statistical Analysis

The results are the mean of 3 values, and measurement data are shown as the mean ± standard deviation (SD). Statistical analysis was carried out using IBM SPSS Win software version 20. Principal component analysis and Pearson’s correlation were executed using Excel Analyze-it 1.56.

## Results

### Evolution of the Physicochemical Parameters

**FIGURE 1:**
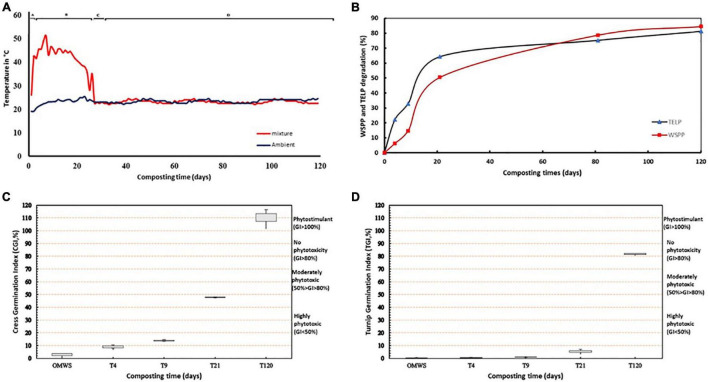
**(A)** Temperature evolution during the composting process. **(B)** Percentage degradation of total extracted lipids (TELP) and Water-soluble polyphenols (WSPP) during composting. **(C,D)** Germination Index (GI%) of cress and turnip, respectively. Seeds were treated with extract of OMWS compost after 4 days (T4), 9 days (T9), 21 days (T21), 52 days (T52), and 121 days (T121) of composting.

The temperature evolution during OMWS-GW co-composting showed a classical dynamic represented by the succession of mesophilic, thermophilic and maturation phases. The ambient temperature of the composted mixture on the first day of this experiment was 23°C (Figure 1A), and after only 2 days, the temperature reached 42°C and then 54°C after 7 days. The thermophilic phase was maintained for 17 days. Between days 17 and 26, the temperature steadily decreased until reaching an ambient value of 23°C, which was maintained until the end of the composting process.

The initial pH value was 5.57, which then increased to 6.5 on the 21st day, corresponding to the end of the cooling phase (Table 2). The final pH value was 8, resulting from the high decomposition rate (DR)% of organic matter, which exceeded 62% after 120 days of composting (Table 2). For the EC, the initial value of the mixture was 2.9 mS cm^–1^, and it then followed the same trend as that of the pH, as it reached 3.8 and 4.2 mS cm^–1^ after 21 and 120 days, respectively.

**TABLE 2:** Physicochemical characteristics of the compost at T0 = initial mixture and after 4 days (T4), 9 days (T9), 21 days (T21), and 120 days (T120).

Composting time (days)	Moisture (%)	TOC (%)	% TKN	pH	EC (ms/cm)	DR (%)	NH^+^_4_/NO_3_^–^
T0	54.2 (0.74)^a^	48.5 (1.03)^a^	0.41 (0.1)^a^	5.6 (0.01)^a^	2.94 (0.01)^a^	–	11.61 (0.4)^a^
T4	50.6 (0.25)^b^	46.23 (0.9)^b^	0.69 (0.1)^b^	5.7 (0.06)^a^	3.3 (0.08)^b^	28.4 (1.3)^a^	4.39 (0.09)^b^
T9	48.6 (0.61)^c^	45.2 (0.5)^bc^	1.03 (0.08)^c^	5.9 (0.05)^b^	3.4 (0.05)^b^	36.3 (3.9)^b^	2.27 (0.2)^c^
T21	47.9 (0.32)^c^	42.2 (0.6)^b^	1.1 (0.01)^c^	6.5 (0.04)^c^	3.82 (0.01)^c^	47.64 (3.2)^c^	1.41 (0.13)^d^
T120	41.5 (0.7)^d^	37.7 (0.6)^c^	1.03 (0.04)^c^	7.97 (0.13)^d^	4.2 (0.15)^bd^	62.32 (0.03)^d^	0.91 (0.03)^d^

*Values are means ± SD of 3 replicates. Numbers in the same column denoted by different letters differ significantly at P < 0.05 according to the Student, Newman–Keuls test.*

The TOC decreased progressively during 120 days of composting (Table 2). The TOC contents decreased from 46.23 to 42.24% after 21 days and then further decreased to 37.7% at the end of the process. The initial nitrogen content (0.41%) significantly increased during the thermophilic phase by 173%. Thereafter, it gradually stabilized at a lower value (1.03%) (Table 2). At the end of the experiment, the NH_4_^–^/NO_3_^–^ ratio decreased from 11.61 to 0.91, indicating that the compost had reached maturity.

### Evolution of Toxicity

Co-composting of OMWS-GW resulted in a significant reduction in polyphenols and lipids, which constitute the main toxic organic compounds in OMWS. The water-soluble polyphenol (WSPP) concentration and total extract lipid (TELP) variations during composting followed a zero-order kinetic model (Figure 1B). The curve fitting of the experimental data gave the following equations:

**WSPP = 0.695t + 11.9014 (R^2^ = 0.853)**

**TELP = 0.5514t + 24.418 (R^2^ = 0.763)**

For both fractions, a steady decrease was registered throughout composting. Two distinct phases were observed: a fast degradation dynamic during the thermophilic phase, which resulted in the elimination of nearly 50% WSPP and 60% TLEP. Then, a slow degradation dynamic until the end of the experiment resulted in a maximum decrease of 81.30 and 84.54% for TLEP and WSPP, respectively. Evaluation of the germination index (GI) showed a positive correlation with TLEP and WSPP degradation dynamics. Indeed, after the 4th day (Figures 1C,D), the GI values were 8.13 and 0.6 for Cress and Turnip, respectively (highly phytotoxic GI < 50%), showing the high toxicity of the compost during the initial composting phase, which was maintained even after the thermophilic phases for the two-studied species, with values of 47 and 4.6% for Cress and Turnip, respectively. However, at the end of the maturation phase, the phytotoxic effects were completely alleviated as the GI reached 110 and 82% for Cress and Turnip, respectively.

### Change in Microbial Diversity During Olive Mill Wastewater Sludge-Green Waste Co-composting

#### Change in the Microbial Communities Through a Culture-Dependent Approach

**FIGURE 2:**
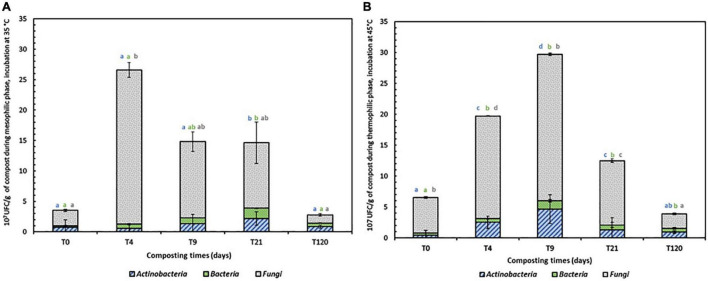
Mesophilic **(A)** and thermophilic **(B)** indigenous microbiota (Actinobacteria, Bacteria and fungi (Fungi = Molds and yeasts) groups during co-composting process T_0_ = initial mixture, T_4_ mixture after 4 days, T_9_ mixture after 9 days, T_21 mixture_ after 21 days and T_120_ mixture after 120 days. Bars represent mean values ± SE of 3 replicates that indicate 95% confidence intervals and columns denoted by a different letter differ significantly at *p* < 0.05.

The evolution of the total mesophilic and thermophilic microbiota during the composting process (Figure 2) was correlated with temperature variation. The total mesophilic microbiota showed a maximum increase at day 4 from 3.53 × 10^7^ to 26.61 × 10^7^. Then, a gradual decrease was observed, and a value of 2.77 × 10^7^ was reached after 4 months of co-composting. For the thermophilic microbiota, the optimal increase in enumerated microorganisms (from a value of 6.54 to 29.7 × 10^6^) was determined at day 9, corresponding to the thermophilic phase. Later, a decrease to 3.9 × 10^6^ by the end of the co-composting process was recorded.

During the biodegradation phase (Figure 2A), the mesophilic microbiota was initially present. Once the temperature surpassed 40°C, thermophilic microorganisms became increasingly dynamic and progressively replaced the mesophilic microorganisms. From days 0 to 9 of the composting process, mesophilic Actinobacteria showed a decrease in total abundance from 16.56 to 9.15%, while thermophilic Actinobacteria showed an increase from 6.88 to 15.72%. In the bacterial community, the total abundance was less than 7% for both categories, with a successive increase from 4.63 to 6.46% and decrease from 5.5 to 4.47% for mesophilic and thermophilic bacteria. However, the fungal abundance presented the highest values during the entire process of composting, with variations of 78.8 to 84.37% and 87.61 to 79.79% for mesophilic and thermophilic fungi, respectively, after 9 days of composting.

By the end of the co-composting process, the abundance of mesophilic and thermophilic fungi decreased from 78.80 and 87.61% to 48.73 and 60%, respectively. Nevertheless, a decrease in Actinobacteria abundancy from 30.69 and 25% to 20.58 and 15% for mesophilic and thermophilic bacteria, respectively, was recorded.

#### Change in Microbial Communities Through Metagenomic Analysis

**FIGURE 3:**
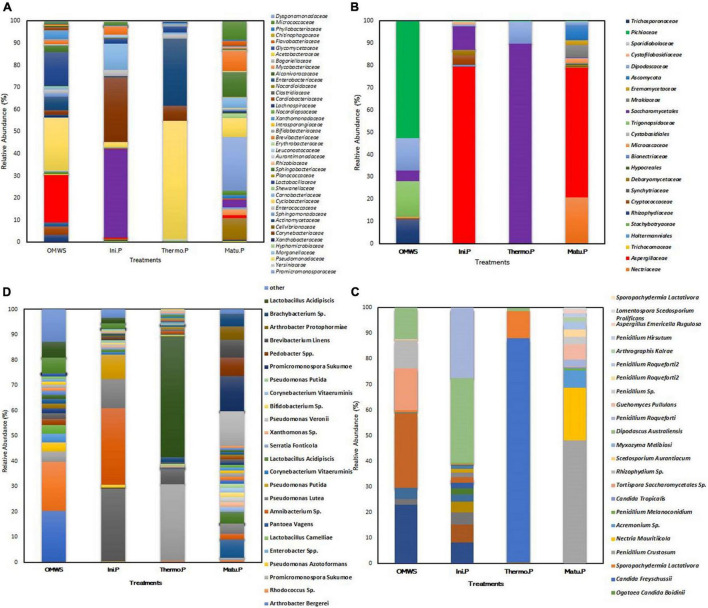
Microbial diversity expressed as relative abundance (%) of OTUs. **(A,B)** Bacterial and fungal families. **(C,D)** Bacterial and fungal species. Raw olive mill waste sludge (OMWS). Initial phase (Ini.P), Thermophilic phase (Thermo.P) and Maturation phase (Matu.P).

Metagenomic analysis of samples representing the key phases of the composting process (mesophilic, thermophilic and maturation) revealed that bacterial taxonomic diversity initially existing in raw OMWS was mostly dominated by Gammaproteobacteria (52.4%), Bacilli (18%) and Actinobacteria (17.8%). At the species level, bacteria were prevalent, namely, *Zymobacter palmae* (20%), *Pseudomonas* sp. (19%), and *Lactobacillus acidipiscis (6%)*. Moreover, fecal bacteria were also identified, such as *Staphylococcus sciuri* (0.003%), *Enterococcus faecalis* (0.1%), *Enterococcus gallinarum* (0.1%), and *Enterococcus raffinosus* (0.003%), which should be considered potential infectious agents. For fungi, diversity analysis revealed that yeasts were the most abundant genus in raw OMWS (Figure 3). These were mostly represented by the *Pichia* genus, with almost 53% of the total identified population. The remaining genera were *Tortispora* (16%), *Dipodascus* (*12%*), *Rhizophydium (11%), Candida* (4.4%), *Galactomyces* (2.4%), and *Scedosporium* (0.4%).

In the initial mixture (Figure 3C), the fungal microbiome was dominated by four species (> 5%), namely, *Dipodascus australiensis* (33.18%), *Penicillium roqueforti* (27.27%), *Pichia* sp. (8.17%) *and Cryptococcus* sp. (7%). Similarly, the bacterial microbiome (Figure 3D) was dominated by *Psychrobacter aquaticus* (30.3%), *Corynebacterium variabile* (28.7%), *Carnobacterium maltaromaticum* (11.5%), and *Psychrobacter pulmonis* (9.7%).

**FIGURE 4:**
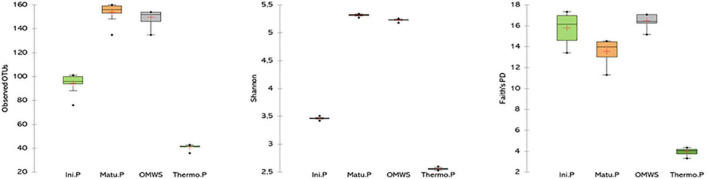
Number of observed OUT’s and distribution of Shannon and Faith’s phylogenetic alpha diversity. Raw olive mill waste sludge (OMWS) Initial phase (Ini.P), Thermophilic phase (Thermo.P) and Maturation phase (Matu.P).

By the end of thermophilic phase, major changes were observed in taxa abundancy (Figure 3C), as the compost was dominated by only two fungal and three bacterial species, namely, *Candida freyschussii* (87.3%), *Sporopachydermia lactativora* (10.6%), *Pseudomonas syringae* (47.8%), *Actinobacter* sp. (30.3%) and *Corynebacterium variabile* (6.1%). After 120 days of compost maturation (Figure 3D), the fungal community was mainly represented by four species, *Penicillium crustosum* (48%), *Nectria mauritiicola* (20.7%), *Acremonium* sp. (6.7%) and *Guehomyces pullulans* (5.9%). The minor representatives (>1%) were dominated by *Penicillium* sp. (2.9%), Penicillium *roqueforti* (2.9%) and *Penicillium hirsutum* (1.6%). On a related note, the bacterial diversity significantly shifted at the end of the maturation phase compared to the initial mixture. Indeed, fecal bacteria were not detected, and the percentage of each bacterial class decreased except for Actinobacteria, for which a threefold increase (60%) was noted compared to raw OMWS. At the species level, the most dominant bacteria were *Promicromonospora sukumoe* (13.9%), *Pseudomonas stutzeri* (7.3%), *Pedobacter* sp. (7.1%), *Brevibacterium linens* (7%) and *Arthrobacter protophormiae* (5.3%), followed by *Isoptericola* sp. (4.6%), *Carnobacterium maltaromaticum* (4%), *Psychrobacter aquaticus* (2.3%) and *Promicromonospora vindobonensis* (2%). Moreover, alpha diversity was determined for important composting phases by calculating the number of OTUs, Shannon index and Faith’s PD (Figure 4). Higher microbial diversity index was recorded for raw OMWS and samples of the compost maturation phase, while the lower diversity was recorded for samples of the thermophilic phase.

## Discussion

### Physicochemical and Toxicity Changes During Green Waste-Olive Mill Wastewater Sludge Cocomposting

#### Evolution of Temperature, pH, and Nitrogen

Temperature evolution during the composting process is related to microbial community changes during the biodegradation of composted wastes, which plays an important role during maturation and stabilization (Ma et al., 2018). The initial increase in pH is attributed to the organic matter richness of the mixture, especially the lipid and phenolic compounds that microorganisms metabolize more quickly, in addition to the low molecular weight organic acids that are volatilized due to the higher temperature of the thermophilic phase (Tsai and Chang, 2019). The final pH value was 8, which is a direct result of the high decomposition rate of organic matter, which exceeded 62% after 120 days of composting, and the elimination of large amounts of secondary metabolites produced by microorganisms during biodegradation, such as acetic acid and butyric acid (Wei et al., 2016). Furthermore, the %TOC decrease during the composting process was attributed to microbial activity, which reduced the organic content in the composted mixture. According to Huang et al. (2019), this effect is related to active thermophilic microorganisms with a great ability to degrade organic carbon and eliminate various organic compounds initially present in the mixture. The enhancement in nitrification might explain the decrease in the NH_4_^–^/NO_3_^–^ ratio to 0.91, indicating that compost reached maturity based on the standard maturation index reported by Barje et al. (2012).

#### Evolution of Toxicity

After 4 months of composting, the total lipids and polyphenols decreased, which is in alignment with the findings of Hachicha et al. (2009), who reported similar results for OMWS/sesame bark cocomposting. Those authors attributed this reduction to the use of those compounds as a carbon source by indigenous microorganisms through the conversion of phenols to simpler structures or quinones through lignolytic enzymes such as laccase and peroxidase, which are supplied during humic substance polymerization (Ait Baddi et al., 2009). Lipid degradation could be explained by the ability of microbes to breakdown several forms of lipids, such as fatty acids and triglycerides.

The use of the germination index (GI) to evaluate compost maturity by studying phytotoxicity risks for plants is acceptable. Compost is exempt from any potential toxicity to plants if the GI values are greater than 80% (Zhang et al., 2020). Several factors have been previously reported to be responsible for inducing phytotoxicity effect, during the OMWS compost evaluation, this effect has been attributed to the presence of several compounds such as organic acids and phenol compounds, impacting negatively the seed germination (Hachicha et al., 2009; Filippi et al., 2013). According to Piotrowska et al. (2006), many seed species are sensitives to phenol presence such as tomato, which are related to their own genotype. In additions to the low pH value, the high concentrations of lipids in OMWS, can negatively influence the growth and biological activities of seeds, which was negatively correlated with GI reduction (Paulino et al., 2006; Hachicha et al., 2009). In agreement with our results, Said-Pullicino et al. (2007), concluded that a value of GI superior to 60%, the toxicity could be considered as well treated. Our results revealed that phytotoxic effects remained even after the thermophilic phase, as the PP and Lp contents were still relevant. However, at the end of the maturation phases, the phytotoxic effects were completely alleviated, as the GI% reached 110 and 82% for Cress and Turnip, respectively. Hence, the occurrence of plausible biostimulatory effects was demonstrated.

#### Microbial Community Structure of the Olive Mill Wastewater Sludge

Microbial diversity analysis showed that raw OMWS was mainly dominated by fungi, which represented nearly 54% of all identified kingdoms (including bacteria). Similar results were recently reported by Martínez-Gallardo et al. (2021), who found that in OMWS sampled from several ponds, the fungal community systematically outnumbered its bacterial counterpart. Furthermore, the same authors revealed that the bacterial community structure of OMWS is highly dependent on physicochemical parameters, notably nutrient content, pH and moisture content. Indeed, *Proteobacteria* were prevalent in OMWS with neutral pH and high moisture. Actinobacteria were dominant in dry/alkaline OMWS, which is in alignment with our results. Notably, physicochemical traits are not the only factors that influence the microbial diversity and functionality of OMWS. According to Tsiamis and Tzagkaraki (2012), the latter are significantly affected by olive cultivars. Those authors investigated the bacterial profile of OMWS generated from various olive varieties and found only 15% similarity in terms of the identified OTUs. Moreover, the cultivation and harvesting practices had a large influence on the microbial community distribution in OMWS. For example, Carlozzi et al. (2015) showed that fermentative bacteria in OMWS resulting from the early collection and harvesting “hand collection” of the *Olea europaea* variety were restricted to the two families *Peptococcaceae* and *Sporolactobacillaceae* (Kavroulakis and Ntougias, 2011). On another note, the very few studies that thoroughly investigated the fungal diversity in OMWS reported very contrasting results. For example, Martínez-Gallardo et al. (2021) revealed that the OMWS fungal community was dominated by genera such as *Fusarium*, *Aspergillus*, *Scopulariopsis*, *Tritirachium*, *Scedosporium*, and *Microascus*, depending on the pond characteristics. Slama et al. (2021) identified four dominant genera, namely, *Nakazawaea*, *Saccharomyces*, *Lachancea*, and *Candida*. Our findings were very different from those previously reported, as the dominant fungal genus was *Pichia*, which constituted half of the identified fungal population, followed by *Tortispora*, *Dipodascus*, and *Candida*. The prevalence of *Pichia* sp. was not surprising, as several studies have reported a strong correlation between the occurrence of *Pichia* species and the detoxification of OMWS (Morillo et al., 2008; Sinigaglia et al., 2010; Arous et al., 2016). Moreover, to our knowledge, this is the first time that the *Diplodocus* genus has been identified in OMWS. This genus was represented solely by the cactophilic *Dipodascus australiensis*, which is not surprising knowing that the pond from where the sample was taken was surrounded by *Opuntia* spp. Similarly, *Tortispora* and *Pichia* are usually associated with decaying cactus tissue (Lachance et al., 1988). These observations suggest that the community structure of OMWS is highly dependent on the faunal and floral specificities of the sampling sites.

#### Changes in Microbial Community Structure During Olive Mill Wastewater Sludge/Green Waste Cocomposting Depend on Physicochemical Traits and Microbe-Microbe Interactions

**FIGURE 5:**
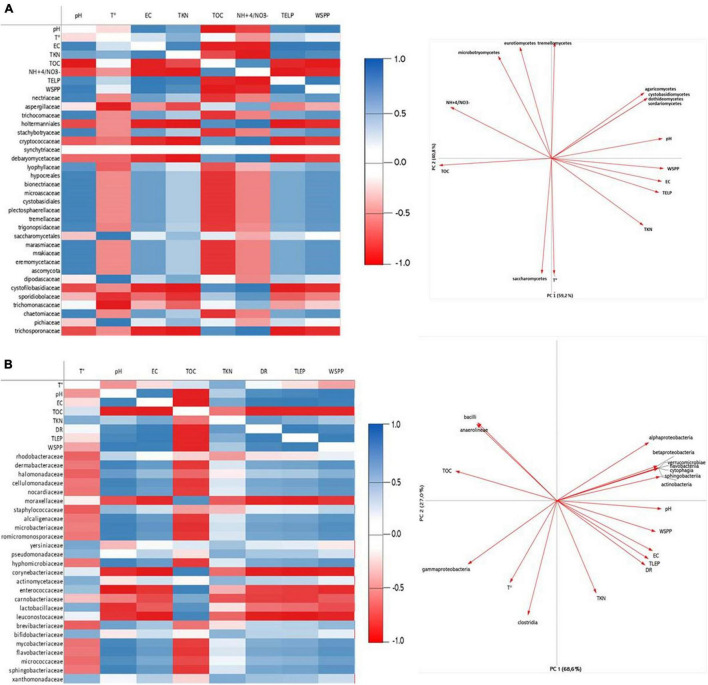
Correlation heatmap and Principal Component Analysis (PCA) of the main parameters during composting treatment: TOC, Total organic carbon; K, potassium; Mn, Manganese; Phenols; TD, Decomposition rate; pH; Ca, Calcium with Fungi **(A)** and Bacteria **(B)** genera diversity.

Adding green waste to OMWS significantly affected the composition of the microbial communities, as the diversity of Actinobacteria increased twofold compared to raw OMWS, which is plausible due to the actinobacterial richness of green waste. However, members of the *Proteobacteria* family (primarily represented by *Moraxellaceae*) were still the most dominant. Similarly, for fungi, the previously dominant *Pichiacea* were greatly outnumbered by *Microascaceae*, which represented more than 70% of the fungal diversity of the initial mixture. At the end of the thermophilic phase, half of the polyphenols and nearly 60% of total lipids were degraded, which was accompanied by a significant change in both fungal and bacterial diversity, suggesting the existence of a significant correlation between microbial changes and pollutant dynamics during the composting process. Principal component and Pearson correlation analyses (Figure 5 and Supplementary Material 1) revealed that overall pH, temperature and EC were the factors that affected most community structures. Moreover, the pace at which those parameters changed during the composting process was paramount, which was demonstrated by the occurrence of fast and slow phases of polyphenol and lipid degradation. Temperature is particularly important, as this is the first factor to undergo significant changes, which combined with PP content, initially shape the compost microbial community. Our study showed that most of the microbial species were negatively affected by temperature increases, thus demonstrating the prevalence of the mesophilic microbiota. For fungi, only three families were positively correlated with temperature, namely, *Saccharomycetaceae*, *Dipodascaceae*, and *Pichiaceae* (Figure 5A).

Surprisingly, those fungal families were not strongly correlated with polyphenol and lipid removal when taking into consideration the whole composting process. However, this does not exclude a plausible involvement in the degradation of those organic compounds at early stages of composting, notably during the fast degradation phase observed in the first several days (Figure 1B). Indeed, the works of Ben Sassi et al. (2006) and Sinigaglia et al. (2010) previously reported the ability of *Pichia* and *Candida* species (*Candida diddensiae*, *Candida ernobii*, *Pichia holstii, and Pichia membranifaciens*) isolated from Moroccan OMWS to degrade PP, such as p-coumaric, caffeic and vanillic acids. Additionally, many other authors have reported that OMWS detoxification can be directly attributed to species of the *Pichia* genus (Morillo et al., 2008; Arous et al., 2016). Similar trends were observed for bacteria, as the only bacterial classes (*Clostridia* and *Gammaproteobacteria*) that were positively correlated with temperature had limited effects on PP and Lp removal, as shown by PCA. More importantly, even if Actinobacteria were negatively affected by temperature, some thermotolerant representatives (Supplementary Material) were likely to be involved in PP and Lp degradation. For example, at the end of the thermophilic phase, the actinobacterial composition of the mixture was dominated by *Corynebacterium* sp., which have been reported as potent PP degraders (Pradeep et al., 2015). Moreover, the highly sensitive *Pseudomonas syringae* constituted more than 50% of the identified bacterial species, which could be explained by the favorable physicochemical conditions, including the decrease in PP content following microbial removal by fungal and actinobacterial species. In fact, Pseudomonadaceae was strongly correlated with *Actinomycetaceae* (0.995), which demonstrates the importance of microbial species interactions during the composting process. Regarding fungi, the end of the thermophilic phase was marked by the prevalence of two species, namely, *Candida freyschussii*, an oleaginous yeast knowing for its ability to produce lipids from glycerol (Amaretti et al., 2012), and *Sporopachydermia*, which was previously reported to be a lipolytic microorganism (Fickers et al., 2005; Agnolucci et al., 2013). Following the thermophilic phase, pH and EC were the most determinant factors influencing microbial community structure, and similar trends were observed for fungi and bacteria, as phyla (Actinobacteria and Ascomycota) that were favored by pH and EC increases were positively correlated with WSPP and TLEP. On a related note, the results of the culture-dependent analysis revealed that fungi were probably more active than bacteria during the thermophilic phase, as they represented more than 70% of all cultivated species. Usually, bacteria constitute the majority of the microorganisms during composting, with a greater number than Actinobacteria and fungi. Our findings revealed opposite results, which could be attributed to the antimicrobial effect of OMWS (richness in monomeric and polymeric phenolic compounds). Additionally, fungi are largely known for being directly involved in biodegradation through the production of polyphenol oxidase during composting (Carraro et al., 2014; Rigane et al., 2015; Steinmetz et al., 2019). Overall, few studies have investigated shifts in microbial communities during the cocomposting of OMWS, and most of them could not identify relevant causative factors (Milanović et al., 2019). In our case, even if relative abundancy lacks the functionality aspect, both correlation analysis and culture-dependent assays clearly demonstrated that pH, temperature, EC, PP content and microbial competition are the main factors affecting microbial succession.

At the species level, *Pichia manshurica* seems to specialize in lipid degradation, and *Candida* sp. and *Galactomyces* sp. are likely to be strongly involved in phenol biodegradation (Supplementary Material 2), which agrees with several previous works (Pinedo-Rivilla et al., 2009; Alves et al., 2014; Karimi and Hassanshahian, 2016; Oliveira et al., 2018). The identified bacterial species revealed that *Lactobacillus acidipiscis* was the agent most correlated with phenolic and lipid biodegradation (Supplementary Material 2), which was previously identified in a work recently done by Uylaşer and Yildiz (2014) and has been identified as a halophilic lactic acid bacterium able to tolerate up to 8% NaCl from black and green olive samples, representing almost 28% of the total identified genus. The same authors showed important lipolytic and pectolytic activity up to 1.09 and 5.29 U ml^–1^, respectively, as well as a positive decarboxylase activity. To the best of our knowledge, this species has never been identified as a direct agent of phenol degradation, despite its high potential, which will be very useful in the detoxification of organic pollutants in olive mill waste. *L. acidipiscis*, first described by Tanasupawat et al. (2000), was found in isolates from fermented fish. It has also been isolated from soy sauce mash (Tanasupawat et al., 2002).

## Conclusion

These findings highlight the distinct impact of the composition and functional microbiota of the OMWS substrate. Fungi were the most dominant taxon of the cultivable microorganisms and were dominated by the genera *Pichia*, *Candida*, and *Galactomyces*. The genus *Lactobacillus* was the most efficient OMWS bacterial taxon with respect to pollutant degradation. Microbial community succession with respect to physicochemical changes during OMWS has been scarcely investigated, and most of the studies could not clearly identify relevant causative factors explaining the prevalence of a specific microbial community during a given composting phase. At the end of the co-composting experiment, polyphenol and lipid content was reduced by 84.54 and 81.30%, respectively, which suggest that the process needs to be further improved as the degradation of contaminants was not fully optimal. Our study combined culture-dependent and metagenomic approaches to identify key microbial species/classes that have significant effect on distinct phases of the composting process. These results are valuable, as they demonstrate that: 1. OMWS composting should be tailored taking into consideration the specifies of the collecting ponds, as the microbial profile is highly dependent on-site characteristics; and 2. An inoculation methodology taking into account both inoculum composition and time of inoculation may plausibly improve the degradation rate of organic pollutants though providing a competitive advantage for key species during composting.

## Data Availability Statement

The metagenomics raw data of the bioproject are available at NCBI under accession number PRJNA784753 (ID: 784753).

## Author Contributions

YB, MH, YO, and KL conceptualized the study. YB, MH, YO, LE, and KL designed the experiments. YB, MH, YO, ME, and KL reviewed the manuscript. YB and KL performed the tatistical data analysis. YZ contributed to investment. All authors agreed to be accountable for the content of the work.

## Conflict of Interest

The authors declare that the research was conducted in the absence of any commercial or financial relationships that could be construed as a potential conflict of interest.

## Publisher’s Note

All claims expressed in this article are solely those of the authors and do not necessarily represent those of their affiliated organizations, or those of the publisher, the editors and the reviewers. Any product that may be evaluated in this article, or claim that may be made by its manufacturer, is not guaranteed or endorsed by the publisher.

## Footnotes

1
www.mrdnalab.com

2
https://www.ncbi.nlm.nih.gov

## References

[B1] Agnolucci M. Cristani C. Battini F. Palla M. Cardelli R. Saviozzi A. (2013). Microbially-enhanced composting of olive mill solid waste (wet husk): Bacterial and fungal community dynamics at industrial pilot and farm level. *Bioresour. Technol.* 134 10–16. 10.1016/j.biortech.2013.02.022 23500553

[B2] Ait Baddi G. Cegarra J. Merlina G. Revel J. C. Hafidi M. (2009). Qualitative and quantitative evolution of polyphenolic compounds during composting of an olive-mill waste-wheat straw mixture. *J. Hazard. Mater.* 165 1119–1123. 10.1016/j.jhazmat.2008.10.102 19070426

[B3] Alves C. T. Ferreira I. C. F. R. Barros L. Silva S. Azeredo J. Henriques M. (2014). Antifungal activity of phenolic compounds identified in flowers from North Eastern Portugal against Candida species. *Future Microbiol.* 9 139–146. 10.2217/fmb.13.147 24571069

[B4] Amaretti A. Raimondi S. Leonardi A. Rossi M. (2012). Candida freyschussii: An oleaginous yeast producing lipids from glycerol. *Chem. Eng. Trans.* 27 139–144. 10.3303/CET1227024

[B5] Arous F. Azabou S. Jaouani A. Zouari-Mechichi H. Nasri M. Mechichi T. (2016). Biosynthesis of single-cell biomass from olive mill wastewater by newly isolated yeasts. *Environ. Sci. Pollut. Res.* 23 6783–6792. 10.1007/s11356-015-5924-2 26662789

[B6] Barbera A. C. Maucieri C. Cavallaro V. Ioppolo A. Spagna G. (2013). Effects of spreading olive mill wastewater on soil properties and crops, a review. *Agric. Water Manag.* 119 43–53. 10.1016/j.agwat.2012.12.009

[B7] Barje F. El Fels L. El Hajjouji H. Amir S. Winterton P. Hafidi M. (2012). Molecular behaviour of humic acid-like substances during co-composting of olive mill waste and the organic part of municipal solid waste. *Int. Biodeterior. Biodegrad.* 74 17–23. 10.1016/j.ibiod.2012.07.004

[B8] Ben Sassi A. Boularbah A. Jaouad A. Walker G. Boussaid A. (2006). A comparison of Olive oil Mill Wastewaters (OMW) from three different processes in Morocco. *Proc. Biochem.* 41 74–78. 10.1016/j.procbio.2005.03.074

[B9] Bouhia Y. Hafidi M. Ouhdouch Y. Boukhari M. E. M. El Zeroual Y. Lyamlouli K. (2021). Effect of the co-application of olive waste-based compost and biochar on soil fertility and Zea mays agrophysiological traits. *Int. J. Recycl. Org. Waste Agric* 2021 111–127. 10.30486/ijrowa.2021.1906342.1115

[B10] Bouhia Y. Lyamlouli K. El Fels L. Youssef Z. Ouhdouch Y. Hafidi M. (2020). Effect of microbial inoculation on lipid and phenols removal during the co − composting of olive mill solid sludge with green waste in bioreactor. *Waste Biomass Valoriz.* 2020 1077–1073. 10.1007/s12649-020-01077-3

[B11] Box J. D. (1993). Investigation of the Folin-Ciocalteau phenol reagent for the determination of polyphenolic substances in natural waters. *Water Res.* 17, 511–525. 10.1016/0043-1354(83)90111-2

[B12] Carlozzi P. Padovani G. Cinelli P. Lazzeri A. (2015). An innovative device to convert olive mill wastewater into a suitable effluent for feeding purple non-sulfur photosynthetic bacteria. *Resources* 4 621–636. 10.3390/resources4030621

[B13] Carraro L. Fasolato L. Montemurro F. Martino M. E. Balzan S. Servili M. (2014). Polyphenols from olive mill waste affect biofilm formation and motility in *Escherichia* coliK-12. *Microb. Biotechnol.* 7 265–275. 10.1111/1751-7915.12119 24628798 PMC3992022

[B14] Chandna P. Nain L. Singh S. Kuhad R. C. (2013). Assessment of bacterial diversity during composting of agricultural byproducts. *BMC Microbiol.* 13:99. 10.1186/1471-2180-13-99 23651653 PMC3651732

[B15] El Fels L. Yedir O. Hafid M. (2015). Use of the co-composting time extract agar to evaluate the microbial community changes during the co-composting of activated sludge and date palm waste. *Int. J. Recycl. Org. Waste Agric*. 4, 95–103. 10.1007/s40093-015-0089-z

[B16] Fickers P. Benetti P. H. Waché Y. Marty A. Mauersberger S. Smit M. S. (2005). Hydrophobic substrate utilisation by the yeast Yarrowia lipolytica, and its potential applications. *FEMS Yeast Res.* 5 527–543. 10.1016/j.femsyr.2004.09.004 15780653

[B17] Filippi C. Bedini S. Levi-Minzi R. Cardelli R. Saviozzi A. (2013). Cocomposting of olive oil mill by-products: Chemical and microbiological evaluations. *Compost Sci. Util.* 10 63–71. 10.1080/1065657X.2002.10702064

[B18] Galliou F. Markakis N. Fountoulakis M. S. Nikolaidis N. Manios T. (2018). Production of organic fertilizer from olive mill wastewater by combining solar greenhouse drying and composting. *Waste Manag.* 75 305–311. 10.1016/j.wasman.2018.01.020 29366800

[B19] Grioui N. Halouani K. Agblevor F. A. (2019). Assessment of upgrading ability and limitations of slow co-pyrolysis: Case of olive mill wastewater sludge / waste tires slow co-pyrolysis. *Waste Manag.* 92 75–88. 10.1016/j.wasman.2019.05.016 31160029

[B20] Hachicha R. Rekik O. Hachicha S. Ferchichi M. Woodward S. Moncef N. (2012). Co-composting of spent coffee ground with olive mill wastewater sludge and poultry manure and effect of Trametes versicolor inoculation on the compost maturity. *Chemosphere* 88 677–682. 10.1016/j.chemosphere.2012.03.053 22537889

[B21] Hachicha S. Cegarra J. Sellami F. Hachicha R. Drira N. Medhioub K. (2009). Elimination of polyphenols toxicity from olive mill wastewater sludge by its co-composting with sesame bark. *J. Hazard. Mater.* 161 1131–1139. 10.1016/j.jhazmat.2008.04.066 18513861

[B22] Hefnawy M. Nagdi O. M. (2014). Microbial Diversity during Composting Cycles of Rice Straw. *Int. J. Sci. Res.* 3 813–820.

[B23] Huang L. Niu G. Feagley S. E. Gu M. (2019). Evaluation of a hardwood biochar and two composts mixes as replacements for a peat-based commercial substrate. *Ind. Crops Prod.* 129 549–560. 10.1016/j.indcrop.2018.12.044

[B24] Hytiris N. Kapellakis I. E. De La Roij R. Tsagarakis K. P. (2004). The potential use of olive mill sludge in solidification process. *Resour. Conserv. Recycl.* 40 129–139. 10.1016/S0921-3449(03)00038-7

[B25] Jarboui R. Sellami F. Azri C. Gharsallah N. Ammar E. (2010). Olive mill wastewater evaporation management using PCA method Case study of natural degradation in stabilization ponds (Sfax. Tunisia). *J. Hazard. Mater.* 176 992–1005. 10.1016/j.jhazmat.2009.11.140 20036054

[B26] Karimi M. Hassanshahian M. (2016). Isolation and characterization of phenol degrading yeasts from wastewater in the coking plant of Zarand. Kerman. *Braz. J. Microbiol.* 47 18–24. 10.1016/j.bjm.2015.11.032 26887222 PMC4822744

[B27] Kavroulakis N. Ntougias S. (2011). Bacterial and b -proteobacterial diversity in Olea europaea var. mastoidis - and O. europaea var. koroneiki -generated olive mill wastewaters: influence of cultivation and harvesting practice on bacterial community structure. *World J. Microbiol. Biotechnol.* 27. 57–66. 10.1007/s11274-010-0426-3

[B28] Kavvadias V. Doula M. K. Komnitsas K. Liakopoulou N. (2010). Disposal of olive oil mill wastes in evaporation ponds: effects on soil properties. *J. Hazard. Mater.* 182 144–155. 10.1016/j.jhazmat.2010.06.007 20580156

[B29] Kavvadias V. Elaiopoulos K. Theocharopoulos S. Soupios P. (2017). Fate of Potential Contaminants Due to Disposal of Olive Mill Wastewaters in Unprotected Evaporation Ponds. *Bull. Environ. Contam. Toxicol.* 98 323–330. 10.1007/s00128-016-1922-4 27663444

[B30] Lachance M.-A. Starm W. T. Phaff H. J. (1988). Identification of yeasts found in decaying cactus tissue. *Can. J. Microbiol.* 1025–1035. 10.1139/m88-181

[B31] Li Z. Yang Y. Xia Y. Wu T. Zhu J. Wang Z. (2019). The succession pattern of bacterial diversity in compost using pig manure mixed with wood chips analyzed by 16S rRNA gene analysis. *bioRxiv.* 2019:674069. 10.1101/674069

[B32] Ma H. K. Pineda A. Van der Wurff A. W. G. Bezemer T. M. (2018). Carry-over effects of soil inoculation on plant growth and health under sequential exposure to soil-borne diseases. *Plant Soil* 433 257–270. 10.1007/s11104-018-3837-9

[B33] Martínez-Gallardo M. R. López M. J. López-González J. A. Jurado M. M. Suárez-Estrella F. Pérez-Murcia M. D. (2021). Microbial communities of the olive mill wastewater sludge stored in evaporation ponds: the resource for sustainable bioremediation. *J. Environ. Manage.* 279:111810. 10.1016/j.jenvman.2020.111810 33341726

[B34] Mekki A. Dhouib A. Sayadi S. (2013). Review: Effects of olive mill wastewater application on soil properties and plants growth. *Int. J. Recycl. Org. Waste Agric.* 2:15. 10.1186/2251-7715-2-15

[B35] Milanović V. Osimani A. Cardinali F. Taccari M. Garofalo C. Clementi F. (2019). Effect of inoculated azotobacteria and Phanerochaete chrysosporium on the composting of olive pomace: microbial community dynamics and phenols evolution. *Sci. Rep.* 9 1–11. 10.1038/s41598-019-53313-z 31740705 PMC6861245

[B36] Montero I. Miranda M. T. Sepúlveda F. J. Arranz J. I. Rojas C. V. Nogales S. (2015). Solar dryer application for olive oil mill wastes. *Energies* 8 14049–14063. 10.3390/en81212415

[B37] Morillo J. A. Aguilera M. Antízar-Ladislao B. Fuentes S. Ramos-Cormenzana A. Russell N. J. (2008). Molecular microbial and chemical investigation of the bioremediation of two-phase olive mill waste using laboratory-scale bioreactors. *Appl. Microbiol. Biotechnol.* 79 309–317. 10.1007/s00253-008-1422-5 18347793

[B38] Oliveira F. Salgado J. M. Pérez-Rodríguez N. Domínguez J. M. Venâncio A. Belo I. (2018). Lipase production by solid-state fermentation of olive pomace in tray-type and pressurized bioreactors. *J. Chem. Technol. Biotechnol.* 93 1312–1319. 10.1002/jctb.5492

[B39] Palaniveloo K. Amran M. A. Norhashim N. A. Mohamad-Fauzi N. Peng-Hui F. Hui-Wen L. (2020). Food waste composting and microbial community structure profiling. *Processes* 8 1–30. 10.3390/pr8060723

[B40] Paulino A. U. LucenaReinaldo F. P. Monteiro J. M. Nunes A. T. (2006). Evaluating two quantitative ethnobotanical techniques. *Ethnobot. Res. Appl.* 4:60. 10.17348/era.4.0.51-60

[B41] Pergola M. Persiani A. Palese A. M. Di Meo V. Pastore V. D’Adamo C. (2018). Composting: the way for a sustainable agriculture. *Appl. Soil Ecol.* 123 744–750. 10.1016/j.apsoil.2017.10.016

[B42] Pinedo-Rivilla C. Aleu J. Collado I. (2009). Pollutants Biodegradation by Fungi. *Curr. Org. Chem.* 13 1194–1214. 10.2174/138527209788921774

[B43] Piotrowska A. Iamarino G. Rao M. A. Gianfreda L. (2006). Short-term effects of olive mill waste water (OMW) on chemical and biochemical properties of a semiarid Mediterranean soil. *Soil Biol. Biochem.* 38 600–610. 10.1016/j.soilbio.2005.06.012

[B44] Pradeep N. V. Anupama S. Navya K. Shalini H. N. Idris M. Hampannavar U. S. (2015). Biological removal of phenol from wastewaters: a mini review. *Appl. Water Sci.* 5 105–112. 10.1007/s13201-014-0176-8

[B45] Rajhi H. Mnif I. Abichou M. Rhouma A. (2018). Assessment and valorization of treated and non-treated olive mill wastewater (OMW) in the dry region. *Int. J. Recycl. Org. Waste Agric.* 7 199–210.

[B46] Rigane H. Chtourou M. Mahmoud I. Ben Medhioub K. Ammar E. (2015). Polyphenolic compounds progress during olive mill wastewater sludge and poultry manure co-composting, and humic substances building (Southeastern Tunisia). *Waste Manag. Res.* 33 73–80. 10.1177/0734242X14559594 25502693

[B47] Sáez J. A. Pérez-Murcia M. D. Vico A. Martínez-Gallardo M. R. Andreu-Rodríguez F. J. López M. J. (2021). Olive mill wastewater-evaporation ponds long term stored: Integrated assessment of in situ bioremediation strategies based on composting and vermicomposting. *J. Hazard. Mater.* 402:123481. 10.1016/j.jhazmat.2020.123481 32736177

[B48] Said-Pullicino D. Erriquens F. G. Gigliotti G. (2007). Changes in the chemical characteristics of water-extractable organic matter during composting and their influence on compost stability and maturity. *Bioresour. Technol.* 98 1822–1831. 10.1016/j.biortech.2006.06.018 16935491

[B49] Sinigaglia M. Di Benedetto N. Bevilacqua A. Corbo M. R. Capece A. Romano P. (2010). Yeasts isolated from olive mill wastewaters from southern Italy: Technological characterization and potential use for phenol removal. *Appl. Microbiol. Biotechnol.* 87 2345–2354. 10.1007/s00253-010-2684-2 20532759

[B50] Slama H. Ben Bouket A. C. Alenezi F. N. Khardani A. Luptakova L. Vallat A. (2021). Applied sciences olive mill and olive pomace evaporation pond’s by-products: toxic level determination and role of indigenous microbiota in toxicity alleviation. *Appl. Sci.* 11:5131.

[B51] Souilem S. El-Abbassi A. Kiai H. (2017). *Olive oil production sector: Environmental effects and sustainability challenges.* Amsterdam: Elsevier Inc, 10.1016/B978-0-12-805314-0.00001-7

[B52] Steinmetz Z. Kurtz M. P. Zubrod J. P. Meyer A. H. Elsner M. Schaumann G. E. (2019). Biodegradation and photooxidation of phenolic compounds in soil—A compound-specific stable isotope approach. *Chemosphere* 230 210–218. 10.1016/j.chemosphere.2019.05.030 31103867

[B53] Tanasupawat S. Shida O. Okada S. Komagata K. (2000). Lactobacillus acidipiscis sp. nov. and Weissella thailandensis sp. nov., isolated from fermented fish in Thailand. *Int. J. Syst. Evol. Microbiol.* 50 1479–1485. 10.1099/00207713-50-4-1479 10939653

[B54] Tanasupawat S. Thongsanit J. Okada S. Komagata K. (2002). Lactic acid bacteria isolated from soy sauce mash in Thailand. *J. Gen. Appl. Microbiol.* 48 201–209. 10.2323/jgam.48.201 12469319

[B55] Tsai C. Chang Y.-F. (2019). Carbon Dynamics and Fertility in Biochar-Amended Soils with Excessive Compost Application. *Agronomy* 9:511.

[B56] Tsiamis G. Tzagkaraki G. (2012). Olive-Mill Wastewater Bacterial Communities Display a Cultivar Specific Profile. *Curr. Microbiol.* 62 197–203. 10.1007/s00284-011-0049-4 22109856

[B57] Uylaşer V. Yildiz G. (2014). The historical development and nutritional importance of olive and olive oil constituted an important part of the mediterranean diet. *Crit Rev Food Sci Nutr*. 54 1092–1101. 10.1080/10408398.2011.626874 24499124

[B58] Walkley A. Black I. A. (1934). An examination of Degtjareff method for determining soil organic matter and a proposed modification of the chromic acid titration method. *Soil Sci.* 37, 29–38. 10.1097/00010694-193401000-00003

[B59] Wei Y. Zhao Y. Wang H. Lu Q. Cao Z. Cui H. (2016). An optimized regulating method for composting phosphorus fractions transformation based on biochar addition and phosphate-solubilizing bacteria inoculation. *Bioresour. Technol.* 221 139–146. 10.1016/j.biortech.2016.09.038 27639232

[B60] Zhang W. Yu C. Wang X. Hai L. Hu J. (2020). Increased abundance of nitrogen fixing bacteria by higher C/N ratio reduces the total losses of N and C in cattle manure and corn stover mix composting. *Waste Manag.* 103 416–425. 10.1016/j.wasman.2020.01.006 31952023

[B61] Zucconi F. Forte M. Monaco A. De Beritodi M. (1981). Biological evaluation of compost maturity. *Biocycle* 22 27–29.

