# Supplementary material for: Microbial Community Succession and Organic Pollutants Removal During Olive Mill Waste Sludge and Green Waste Co-composting
Source: Front Microbiol. 2022 Feb 21;12:814553. doi: 10.3389/fmicb.2021.814553 (PMC8899611; doi:10.3389/fmicb.2021.814553)
Supplement: Supplementary file 1 [file Data_Sheet_1.ZIP › S2.docx]

**
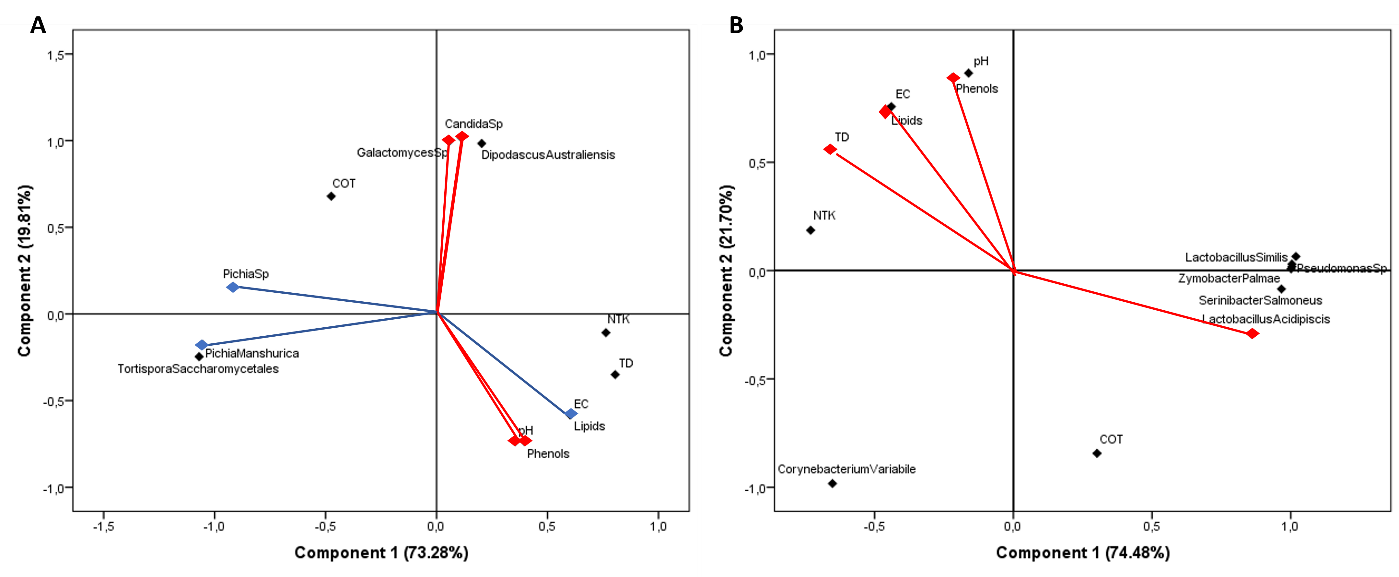
**

**S2:** Principal component analyses (PCA) of the main parameters during composting treatment: TOC, Total organic carbon; K, potassium; Mn, Manganese; Phenols; TD, Decomposition rate; pH; Ca, Calcium and OMWS microbial species (A) fungi and (B) Bacteria
